# Estrogen-induced compensatory mechanisms protect IL-10-deficient mice from developing EAE

**DOI:** 10.1186/s12974-019-1588-z

**Published:** 2019-10-29

**Authors:** Hilary A. Seifert, Grant Gerstner, Gail Kent, Arthur A. Vandenbark, Halina Offner

**Affiliations:** 1 Department of Neurology, Oregon Health & Science University, Portland, OR USA; 2 Neuroimmunology Research, VA Portland Health Care System, R&D-31, 3710 SW US Veterans Hospital Rd., Portland, OR 97239 USA; 3 Department of Molecular Microbiology & Immunology, Oregon Health & Science University, Portland, OR USA; 4 Department of Anesthesiology & Perioperative Medicine, Oregon Health & Science University, Portland, OR USA

**Keywords:** Estrogen (E2), PD-1 ligands, Regulatory B and T cells, IL-10, EAE inhibition

## Abstract

**Background:**

IL-10 knockout (KO) mice are protected from experimental autoimmune encephalomyelitis (EAE) with low-dose estrogen (E2) treatment similar to wild-type (WT) mice. Previous studies have demonstrated a decrease in tumor necrosis factor in all E2-treated groups, which led to the protection of the mice.

**Methods:**

This study used IL-10 KO mice and WT mice treated either with E2 or sham pellets 7 days prior to induction of EAE. Mice were observed for 21 days post-immunization. The spleen, inguinal lymph nodes, and brain were evaluated by flow cytometry. Spinal cords were evaluated using a cytokine/chemokine array, RT-PCR, and histology.

**Results:**

This study demonstrates that E2 treatment induced three heightened regulatory mechanisms that potentially protect IL-10 KO mice from EAE: (1) an increase in programmed death-ligands 1 and 2 on monocytes and macrophages in the periphery and within the CNS; (2) an increase in CD73 in the inflamed CNS, which can increase the production of the anti-inflammatory molecule adenosine; and (3) a decrease in CD4^+^CD25^+^FoxP3^+^ regulatory T cells in the spleen. Together, these factors comprise an alternative compensatory mechanism that significantly downregulates key pro-inflammatory cytokine, chemokine, and chemokine receptor genes which are enhanced in the spinal cord of IL-10 KO mice. This group of E2-treated mice remained asymptomatic after EAE challenge similar to E2-treated WT mice, despite their having more T and B lymphocytes in the brain, and modestly increased demyelination in the spinal cord.

**Conclusion:**

These results indicate that previously unrecognized compensatory mechanisms of EAE protection are stimulated by E2 in the absence of IL-10, which can provide disease protection comparable to the IL-10-dependent effects induced by E2 in WT mice.

## Background

Multiple sclerosis (MS) is a chronic autoimmune disease of the CNS. Individuals with MS have demyelinating CNS lesions that lead to various neurologic symptoms including motor dysfunction, sensory disturbances, and cognitive impairments. MS has a higher predominance in females [1]. MS subjects often demonstrate an improvement in clinical symptoms during pregnancy, followed by relapse post-partum [2–4]. This has generated an interest in how different hormones, particularly estrogen, might be able to regulate the immune system and contribute to repair and protection against neural damage. Estrogen is known to modulate the innate and adaptive immune system [5]. Studies in MS subjects have demonstrated low levels of sex hormones, like estrogen, are associated with higher circulating levels of tumor necrosis factor (TNF) and interferon gamma (IFNγ) [6]. Models of experimental autoimmune encephalomyelitis (EAE) have shown that treatment with a low dose of 17β-estradiol (E2) protects against disease development [7, 8] and medium-dose estrogen protects ovariectomized mice from developing EAE [9]. While E2 does not directly affect encephalitogenic T cells [10], it does have a specific effect on antigen-presenting cells, including macrophages, B cells, and dendritic cells which contribute to the downregulation of T cells. E2 can increase T regulatory (Treg) cells and enhance the expression of programmed death receptor 1 (PD-1) [11]. E2 has also been shown to work through estrogen receptor alpha (ERα) [12], and ERα was only important on hematopoietic cells and not endothelial cells to mediate its anti-inflammatory effects [13]. ERα has also been shown to be the primary receptor responsible for protection from EAE [14]. Additionally, female and male mice are protected from EAE with E2 treatment that modulates the immune system through common regulatory pathways to protect both sexes [15].

Initially, B cells were thought to play a pathogenic role in EAE [16–19]. Though B cells are not essential to induce EAE in mice, myelin oligodendrocyte glycoprotein 35–55 (MOG) specific antibodies can enhance inflammation and increase demyelination in EAE [20, 21]. Also, MOG-specific B cells have been shown to act as antigen-presenting cells (APC) in EAE [22]. Additional studies in EAE examining B cell function have revealed a different role for B cells in EAE progression. B cell-deficient (μMT^−/−^) or B cell-depleted (anti-CD20 antibody) mice develop a more severe form of EAE compared to WT or naive mice [23, 24]. Regulatory B cells (Breg) have been widely studied in EAE [18, 24–28]. A potent, yet small, population of Breg cells, B10 cells, can protect mice from EAE, and this protection is associated with B10 production of interleukin-10 (IL-10) [28]. Other studies have demonstrated that Breg cells are induced with E2 treatment, and this protection is partly dependent on programmed death-ligand 1 (PD-L1) [27]. Additionally, Breg cells treated with E2 can partially prevent μMT^−/−^ mice from developing severe EAE, although significant E2-induced protection still occurred without the addition of the Breg cells [24]. We have also shown that Breg cells can polarize microglia/macrophages towards an anti-inflammatory phenotype with E2 treatment and that polarized microglia/macrophages can influence the transition of naive B cells into Breg cells. Moreover, anti-inflammatory microglia/macrophages induced significantly more IL-10-secreting Breg cells than pro-inflammatory polarized cells or non-polarized cells [25]. Other laboratories have demonstrated that treatment with CpG primed pro-B cells can protect mice from active EAE by developing into Breg cells [29]. Breg cells have also been shown to induce IL-10-producing Treg cells in vivo and to protect mice from active EAE. Breg cells were found in the spleen and mesenteric lymph nodes, while Treg cells were found in the CNS. Finally, there was a reduction in both B cells and T cells in the Breg-treated mice [30].

IL-10 is a potent anti-inflammatory cytokine produced by several immune cell types that can influence the differentiation of T helper (Th) cells, inhibit Th_1_ and Th_17_ cells, inhibit major histocompatibility complex (MHC) class II expression, and increase antibody production by B cells [31]. In untreated individuals with MS, IL-10 levels are lower than healthy controls. When individuals are treated with interferon beta 1 beta (IFNβ), they have increased levels of IL-10 compared to untreated MS subjects [32]. EAE is also more severe in IL-10 knockout (KO) mice compared to wild-type (WT) mice, whereas mice that overexpress IL-10 are protected from developing EAE [33, 34].

PD-1 is a receptor that binds to one of two ligands: programmed death-ligand 1 and 2 (PD-L1 and PD-L2). One of the major roles of PD-1 is to inhibit T cells and create an immunoregulatory environment. This immune mechanism is how many cancers evade immune detection and is able to manipulate the immune system [35, 36]. A new class of cancer drugs targets this mechanism by inhibiting or blocking PD-1 or PD-L1, which allows the immune system, particularly T cells, to attack tumors [37]. In autoimmune diseases, the PD-1/PD-L1 pathway is disrupted, leading to a lack of immune regulation through Tregs. Furthermore, autoreactive T cells are allowed to survive, thus contributing to the ongoing disease processes [38]. Dendritic cells, and B cells to a lesser extent, were found to express PD-L1 that interacted with follicular T helper cells in EAE [39]. In addition to E2 increasing PD-L1 on Breg cells, others have demonstrated that treatment with anti-CD20 antibody depletes B cells during EAE, resulting in a surviving population of antibody-producing B cells with high levels of PD-L1 expression [40]. Additionally, PD-L1 is upregulated on microglia at the peak of EAE disease just prior to the remission phase of the disease. Infiltrating T cells in the CNS also had increased levels of PD-1 at the peak of EAE disease [41].

Treatment with E2 protected mice from developing EAE. However, PD-1 KO mice were not protected from EAE by treatment with E2, suggesting one or both of its ligands are critical for E2-mediated protection from EAE [42]. Further studies have demonstrated that while PD-L2 KO mice were protected from EAE with E2 treatment, the same protection was not observed in PD-L1 KO mice. This suggests PD-L1 is the predominant PD-1 ligand involved in E2 protection during EAE [43]. Regulatory lymphocytes, particularly Bregs that secrete IL-10, are also key to E2 protection from EAE [24]. However, IL-10 KO mice are protected from EAE with E2 treatment. Previous studies have shown IL-10 KO mice, similar to WT mice, downregulate tumor necrosis factor (TNF) with E2 treatment during EAE [8]. Subsequent studies in E2-treated WT mice demonstrated an increase in the PD-1/PD-L pathway and links IL-10 production with IL-10-secreting Bregs [43].

The recent interest in the PD-1/PD-L1 pathway suggests it is a compensatory mechanism which mediates E2 protection against EAE in IL-10 KO mice. In the current study, we demonstrate IL-10 KO mice develop severe EAE and that E2 protects these mice, as previously demonstrated with a significant downregulation of TNF [8]. Additionally, we show here that E2 treatment of IL-10 KO mice significantly increases activity in the PD-L1/2 pathway and increases CD73 expression. These mechanisms are both IL-10 independent immunomodulatory mechanisms. IL-10 KO mice treated with E2 also downregulate several pro-inflammatory cytokines and chemokines not implicated in EAE of WT mice.

## Methods

### Animals

Female wild-type C57BL/6 mice and female IL-10 KO (B6.129P2-*Il10*^*tm1Cgn*^/J) mice (8–10 weeks old) were purchased from The Jackson Laboratory (Sacramento, CA, USA). Mice were housed together by strain and treatment group. Mice were given access to food and water ad libitum and kept on a 12 h light/dark cycle. This study was conducted in accordance with NIH guidelines for the use of experimental animals and the VAPORHCS Animal Care and Use Committee approved protocols.

### E2 pellet implantation and induction of EAE

All mice were treated and immunized as previously described [8]. Briefly, mice were implanted subcutaneously with 2.5 mg/60-day release 17β-estradiol pellets (Innovative Research of America, Sarasota, FL, USA) or sham-treated 1 week prior to immunization. The 2.5 mg E2 pellet produces 1500–2000 pg/ml of E2 in the serum, which is equivalent to pregnancy serum levels of E2 (5000–10,000 pg/ml) [7]. Mice were then immunized with 200 μg mouse MOG-35–55 peptide (PolyPeptide Laboratories, San Diego, CA, USA) in 400 μg Complete Freund’s adjuvant [Incomplete Freund’s adjuvant (IFA, Sigma-Aldrich, St. Louis, MO, USA)] with heat-killed *Mycobacterium tuberculosis* (Mtb, Difco, Detroit, MI, USA) subcutaneously along the flanks at four sites. Additionally, mice were administered 75 ng of pertussis toxin (Ptx, List Biologicals, Campbell, CA, USA) via an intraperitoneal (i.p.) injection on the day of immunization and 200 ng i.p. 2 days later. All mice were monitored daily for weight loss and clinical signs of EAE disease. Mice were scored using the following scale: 0 = normal; 1 = limp tail or mild hind limb weakness; 2 = moderate hind limb weakness or mild ataxia; 3 = moderately severe hind limb weakness; 4 = severe hind limb weakness or mild forelimb weakness or moderate ataxia; 5 = paraplegia with no more than moderate forelimb weakness; and 6 = paraplegia with severe forelimb weakness or severe ataxia or moribund condition. The cumulative disease index (CDI) is the sum of the daily score for each mouse from day 8 to day 21 post-immunization.

### Leukocyte preparation from the spleen, inguinal lymph nodes, and brain

All tissues were collected from mice 21 days post-immunization. Spleens were passed through a 100-μm nylon mesh filter (BD Falcon, Bedford, MA, USA) into RPMI 1640 to create a single cell suspension. Red cells were lysed with 1X Red Cell Lysis Buffer (eBioscience, Inc., San Diego, CA, USA) and the cell suspension subsequently washed with RPMI 1640. Cells were then counted on a Cellometer Auto T4 cell counter (Nexcelom, Lawrence, MA, USA). After counting, cells were centrifuged and resuspended in staining buffer (PBS with 0.1% NaN_3_ and 1% BSA) for staining.

Inguinal lymph nodes (LN) were processed by passing LN through a 100-μm nylon mesh filter (BD Falcon), washing the cells with RPMI 1640, and counted. After centrifugation, cells were resuspended in staining buffer for FACS analysis.

Brains were passed through 100-μm mesh screens and washed as stated above. Cells were resuspended in 80% Percoll (GE Healthcare, Pittsburgh, PA, USA) then overlaid with 40% Percoll to establish a density gradient and centrifuged at 1600 rpm for 30 min following a method previously described [44]. Leukocytes were collected from the resultant interface, counted, and resuspended in staining buffer for staining.

### Flow cytometry

Cells were resuspended at a concentration of 1 × 10^6^ cells/ml in staining buffer. All cells were stained for extracellular markers after being blocked with rat anti-mouse CD16/CD32 Mouse BD Fc Block™ (BD Bioscience, San Jose, CA, USA). After blocking, cells were incubated with fluorescently tagged antibodies and protected from light. The cell viability dye 7-amino-actinomycin D (7AAD) was used to assess cell survival. Cells used for intracellular staining or transcription factor staining were fixed with 4% paraformaldehyde and washed. Intracellular staining was done by resuspending cells in permeabilization buffer (BD Bioscience) and then incubated with antibodies or isotype controls. Transcription factor staining (FoxP3, T-bet, and RORγ) was done with fixation/permeabilization reagents per the manufacturer’s instructions (eBioscience). All samples were then run on a BD Accuri™ C6 (BD Bioscience) with a four-color (FITC, PE, PerCP Cy5.5, and APC) fluorescence flow cytometry analysis.

The following antibodies were used: CD11b (M1/70), CD19 (1D3), CD8 (53-6.7), CD1d (1B1), CD138 (281-2), CD25 (PC61), CD86 (GL1), CD206 (CO68C2), CD122 (TM-β1), CD69 (H1.2F3) (BD Biosciences), CD4 (RM 4-5), PD-L2 (TY25), CD45 (30-F11) (BD Pharmagin), CD44 (1 M7), FoxP3 (FJK-16 s), RORγ (AFKJS-9), PD-1 (RMP1-30) (eBioscience), CD5 (53-7.3), T-bet (4B10), PD-L1 (10F9G2), CD73 (TY/11.8) (Biolegend), and ARG1 (R&D Systems, Minneapolis, MN, USA).

### Histology

Mice were perfused with sterile 1× PBS and the spinal column was removed and placed overnight in 4% PFA at 4 °C. Spinal cords were then dissected from the vertebrae and placed in 70% ethanol. The lumbar sections were embedded in paraffin and cut into 10-μm sections that were stained with Luxol Fast Blue/periodic acid-Schiff/hematoxylin. Stained slides were imaged with light microscopy. ImageJ was used to analyze demyelination and the percentage of nucleated cells in the white matter.

### RNA isolation

RNA was isolated from spinal cords using the RNeasy Mini Kit (Qiagen, Valencia, CA, USA) according to the manufacturer’s protocol. Spinal cords were weighed and suspended in 10 μl/mg of RLT buffer. Three hundred microliters of each sample was diluted 1:2 with RLT for a 30 mg/600 μl mixture. Ten microliters per milliliter of BME was added to the samples. Spinal cords were then homogenized by gentle pipetting. Spinal cord lysates were transferred to QIAshredder tubes and centrifuged at 13,000 rpm for 15 s. Six hundred microliters of 70% alcohol was added to each QIAshredder, spun at 13,000 rpm for 15 s, and transferred to separate RNAeasy columns. The RNAeasy columns were centrifuged twice for 15 s at 13,000 rpm, discarding the flow-through after each step. Seven hundred microliters of Buffer wash RW1 were added to each column. Samples were spun 15 s at 13,000 rpm, with the flow-through discarded. Two successive washes of 500 μl of Buffer RPE were added to the samples and spun at 13,000 rpm for 2 min. The columns were placed in collection tubes and then washed with 50 μl of RNase-free water to elute the RNA. RNA quantity (nanograms/microliter) and quality (A260/280) were measured using a NanoDrop™ One/OneC Microvolume UV-Vis Spectrophotometer (Thermo Scientific, Waltham, MA, USA).

### cDNA synthesis

cDNA was synthesized using the RT^2^ First Strand Kit (Qiagen) using the manufacturer’s protocol. Recommended amounts of Buffer GE, Buffer BC3, Control P2, Reverse Transcriptase Mix, and RNase-free water were used. The samples were incubated at 42 °C for 15 min, then 95 °C for 5 min. RNase-free water was added to each sample.

### Mouse inflammatory cytokine and receptor array

cDNA from three spinal cords per group were pooled for the array. cDNA mix was added to RT^2^ SYBR Green ROX qPCR Mastermix (Qiagen), along with RNase-free water. The master mixes were loaded into each well of RT^2^ Profiler™ PCR Array Mouse Inflammatory Cytokines & Receptors (Qiagen) and run on the Applied Biosystems StepOnePlus Real-Time PCR System. mRNA expression was normalized using the expression of various housekeeping genes and compared to the data from the control group according to the 2^−DDCT^ method [45]. Significant results were confirmed with RT-PCR on individual samples. The protocol and analyses were performed according to the manufacturer’s instructions.

### Statistics

Data were analyzed using Prism software (GraphPad Software, La Jolla, CA, USA) using the Mann-Whitney *U* test for determining significance for disease course. All other data were analyzed using ANOVA with a Fisher’s least significant difference post hoc test or Student’s *t* test when appropriate. A *p* value of ≤ 0.05 was considered significant. Data are represented as mean ± standard error of the mean (SEM). All analyses were carried out in blinded fashion.

## Results

### E2 treatment protects IL-10 KO mice from developing severe EAE

**Fig. 1:**
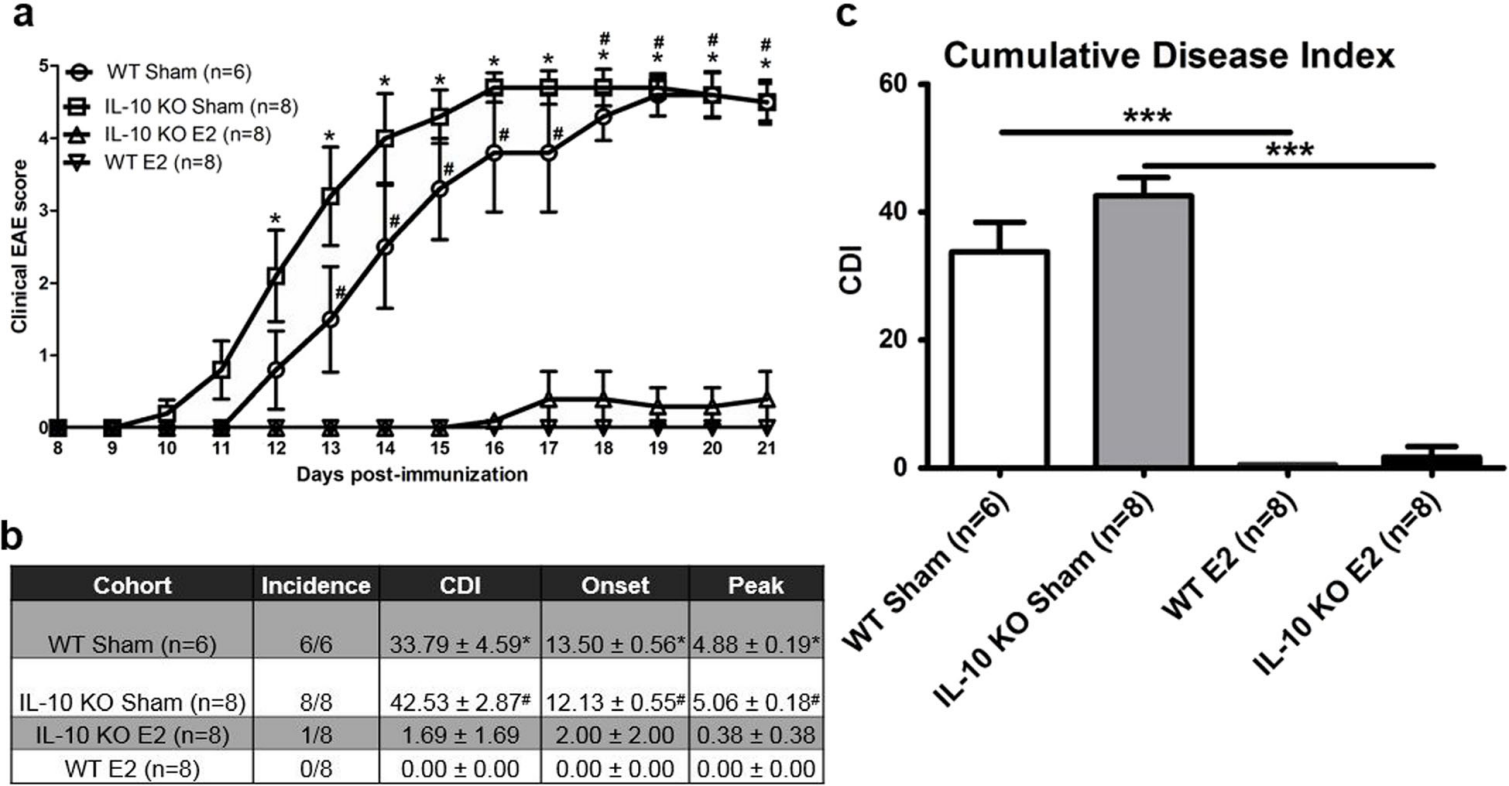
E2 significantly protects WT and IL-10 KO mice from developing EAE. Pretreating mice for 7 days prior to EAE induction with E2 significantly protected WT and IL-10 KO mice from developing severe EAE (*p < 0.05 WT sham vs WT E2 and ^#^*p* < 0.05 IL-10 KO sham vs IL-10 KO E2; **a**). The incidence, disease onset, and peak disease scores were all lower in the E2-treated WT (*n* = 8) and IL-10 KO (*n* = 8) mice compared to sham-treated WT (*n* = 6) and IL-10 KO (*n* = 8) mice, respectively (*p < 0.05 WT sham vs WT E2 and ^#^*p* < 0.05 IL-10 KO sham vs IL-10 KO E2; **b**). Clinical disease scores were significantly lower starting at day 12 post-immunization in WT mice and day 13 post-immunization in IL-10 KO mice, remaining significantly lower through day 21 post-immunization (*p* < 0.05). The cumulative disease index (CDI) was also significantly lower in E2-treated mice compared to the sham-treated mice (WT 0 vs 33.79 ± 4.59; *p* < 0.001 and IL-10 KO 1.69 ± 1.69 vs 42.53 ± 2.87; *p* > 0.001; **c**). Data are represented as mean ± SEM

We treated IL-10 KO mice and WT mice, as controls, with E2 or sham pellets 7 days prior to the induction of EAE and observed the mice for 21 days post-immunization in order to determine the effects of estrogen on the course of EAE in IL-10 KO mice. Treating mice 7 days prior to EAE induction with E2 significantly protected WT and IL-10 KO mice from developing severe EAE, a finding consistent with previous work in our lab [8]. Clinical disease scores were significantly lower starting at day 12 post-immunization for WT mice and day 13 post-immunization for the IL-10 KO mice, remaining significantly lower through day 21 post-immunization (Fig. 1a). The incidence, disease onset, and peak disease scores were all lower in the E2-treated mice compared to sham-treated mice (Fig. 1b). The results were not significantly different between WT and IL-10 KO mice within their respective treatment groups. The CDI was significantly lower in IL-10 KO E2-treated mice compared to IL-10 KO sham-treated mice. The CDI was also significantly lower in WT E2-treated mice compared to WT sham-treated mice (Fig. 1c).

### PD-1 ligand expressions are increased on CD11b^+^ cells in the spleen and LN in E2-treated IL-10 KO mice

**Fig. 2:**
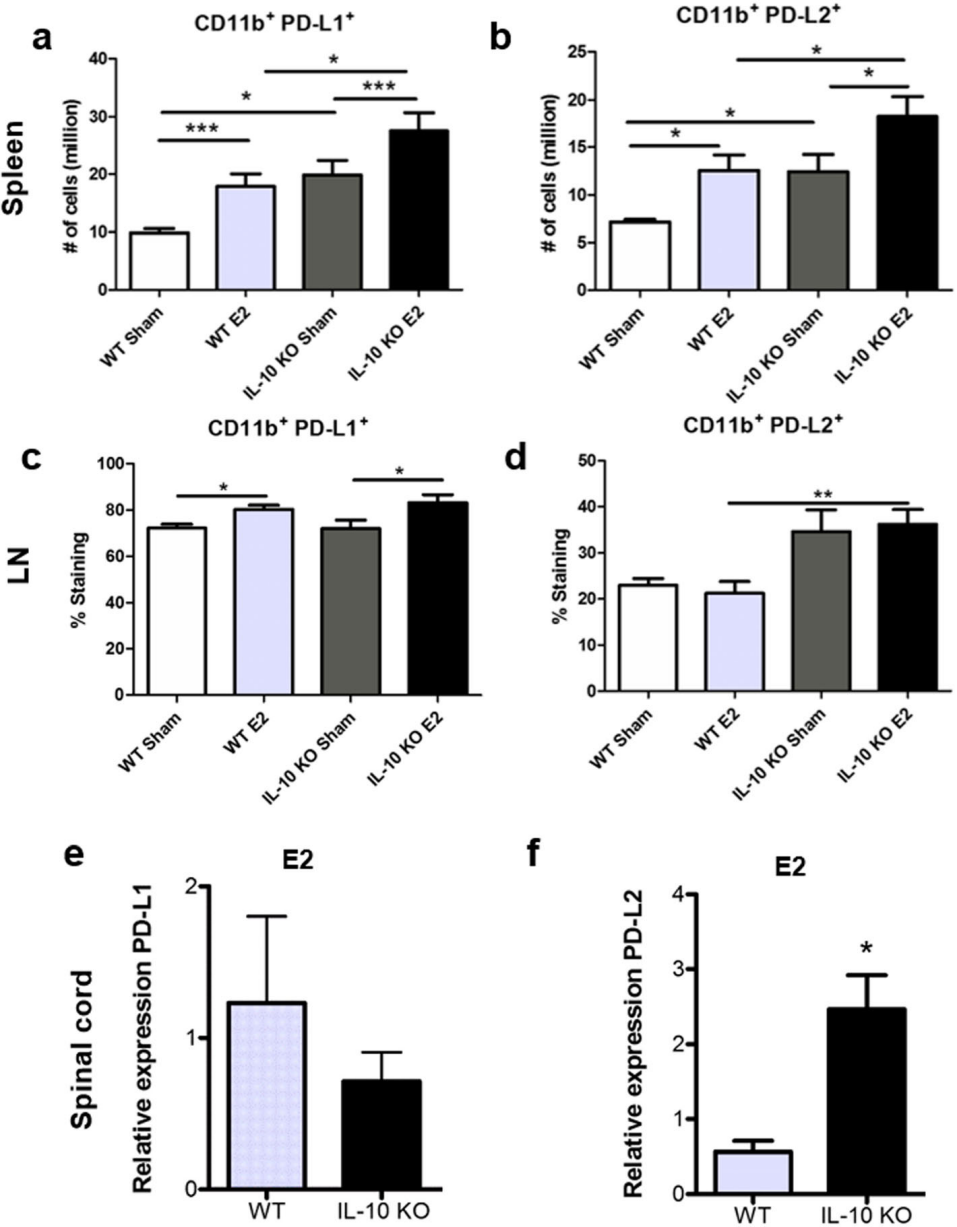
E2-treated IL-10 KO mice increase PD-1 ligand expression on CD11b^+^ cells in the spleen and LN. E2 treatment resulted in a significant increase in the number of PD-L1-expressing CD11b^+^ cells in the spleen in both WT and IL-10 KO mice compared to sham (*p* < 0.001) and significantly increased the number of PD-L1-expressing CD11b^+^ cells in IL-10 KO mice (*n* = 8 for both groups) compared to WT sham (*n* = 6) and E2-treated (*n* = 8) groups, (*p* < 0.05; **a**). PD-L1 frequency was also significantly increased in E2-treated groups compared to sham-treated groups for WT (*n* = 6 and *n* = 8, respectively) and IL-10 KO mice (*n* = 8 for both groups) in the inguinal lymph nodes (*p* < 0.05; **b**). The number of PD-L2-expressing CD11b^+^ cells was significantly increased in the E2-treated groups compared to the sham-treated groups for both WT (*n* = 6 and *n* = 8, respectively) and IL-10 KO (*n* = 8 for both groups) in the spleen (*p* < 0.05). PD-L2-expressing CD11b^+^ cells were also significantly increased in the IL-10 KO mice compared to WT mice for both treatment groups (*p* < 0.05; **c**). In the inguinal lymph nodes, the frequencies of PD-L2 on CD11b^+^ cells were significantly increased in the IL-10 KO E2-treated mice (*n* = 8) compared to the WT-treated mice (*n* = 8; *p* < 0.05; **d**). Additionally, IL-10 KO mice (*n* = 3) treated with E2 have no significant differences in gene expression of PD-L1 in the spinal cord compared to E2-treated WT mice (*n* = 3; **e**). IL-10 KO mice treated with E2 have significantly higher gene expression of PD-L2 (*p* < 0.05) in the spinal cord compared to WT mice treated with E2 (**f**). Data are represented as mean ± SEM

PD-L1 and PD-L2 were significantly increased on CD11b^+^ cells in the spleen in E2 compared to sham-treated WT and IL-10 KO mice and were significantly increased in IL-10 KO E2-treated mice compared to WT E2-treated mice (Fig. 2a, b), respectively. In the inguinal lymph nodes, the frequency of PD-L1 expression on CD11b^+^ cells was significantly increased in E2-treated mice, both WT and IL-10 KO (Fig. 2c). The frequency of PD-L2 on CD11b^+^ cells was only significantly increased in the E2-treated IL-10 KO mice compared to the WT E2-treated mice (Fig. 2d). Additionally, PD-L1 gene expression levels were not significantly different in the spinal cords in IL-10 KO E2-treated mice compared to WT E2-treated mice (Fig. 2e). However, PD-L2 gene expression levels were significantly increased in the spinal cords in IL-10 KO E2-treated mice compared to WT E2-treated mice (Fig. 2f).

### CD73 expression is increased on splenic Breg cells in IL-10 KO mice and is only increased in E2-treated IL-10 KO mice in the CNS

**Fig. 3:**
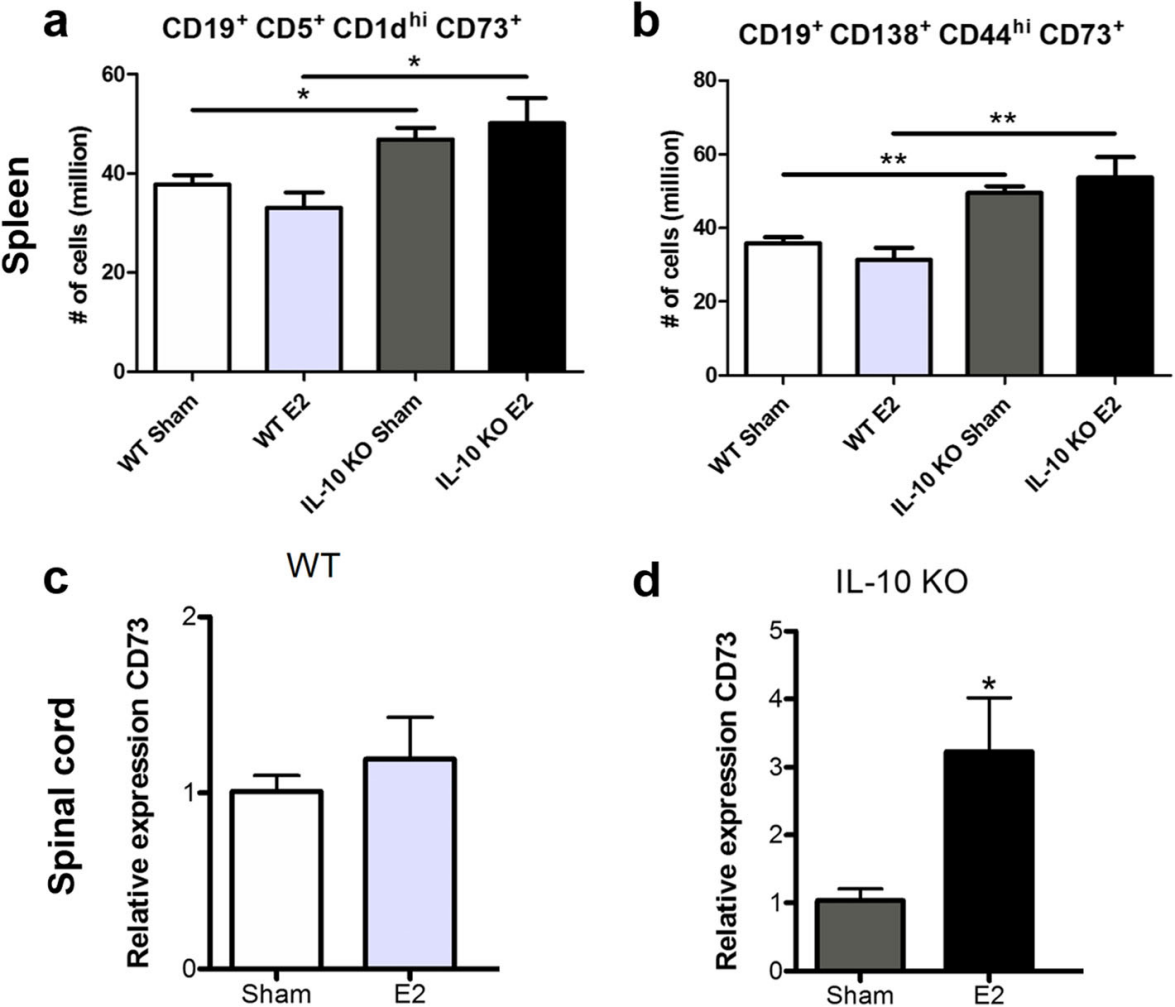
CD73 expression on B_reg_ cells in the spleen and CD73 expression in the CNS. In the spleen, the number of B10 (CD19^+^CD5^+^CD1d^hi^) Breg cells expressing CD73 is significantly increased in both the sham- and E2-treated IL-10 KO groups (*n* = 4), compared to the WT (*n* = 4) groups (*p* < 0.05; **a**). Another subset of Breg cells and plasmablasts (CD19^+^CD138^+^CD44^hi^) had significantly more cells that were expressing CD73 in the spleens of IL-10 KO (*n* = 4) mice in both the sham and E2 treatment groups compared to WT (*n* = 4) mice (*p* < 0.01; **b**). Within the spinal cords, there was not a significant difference in CD73 expression between WT mice regardless of treatment (*n* = 3; **c**). CD73 expression was significantly increased in the spinal cords of E2-treated IL-10 KO mice compared to sham-treated IL-10 KO mice (*p* < 0.05; *n* = 3; **d**). Data are represented as mean ± SEM

The regulatory B cell subsets B10 cells (CD19^+^CD5^+^CD1d^hi^, Fig. 3a) and plasmablasts (CD19^+^CD138^+^CD44^hi^, Fig. 3b) from the spleen significantly increased the number of cells expressing CD73 in the IL-10 KO mice compared to WT mice, independent of estrogen treatment. CD73 is an IL-10 independent immune regulatory receptor pathway found on Breg cells and other cells that upregulates adenosine production [46]. In the spinal cords of E2-treated WT mice, there was not a significant difference in the expression of CD73 compared to sham-treated WT mice (Fig. 3c). However, there was a significant increase in CD73 expression in the spinal cords of E2-treated IL-10 KO mice compared to sham-treated IL-10 KO mice (Fig. 3d).

### Activated splenic CD4^+^CD25^+^ T cells are increased but FoxP3^+^ regulatory T cells are decreased in E2-treated IL-10 KO mice

**Fig. 4:**
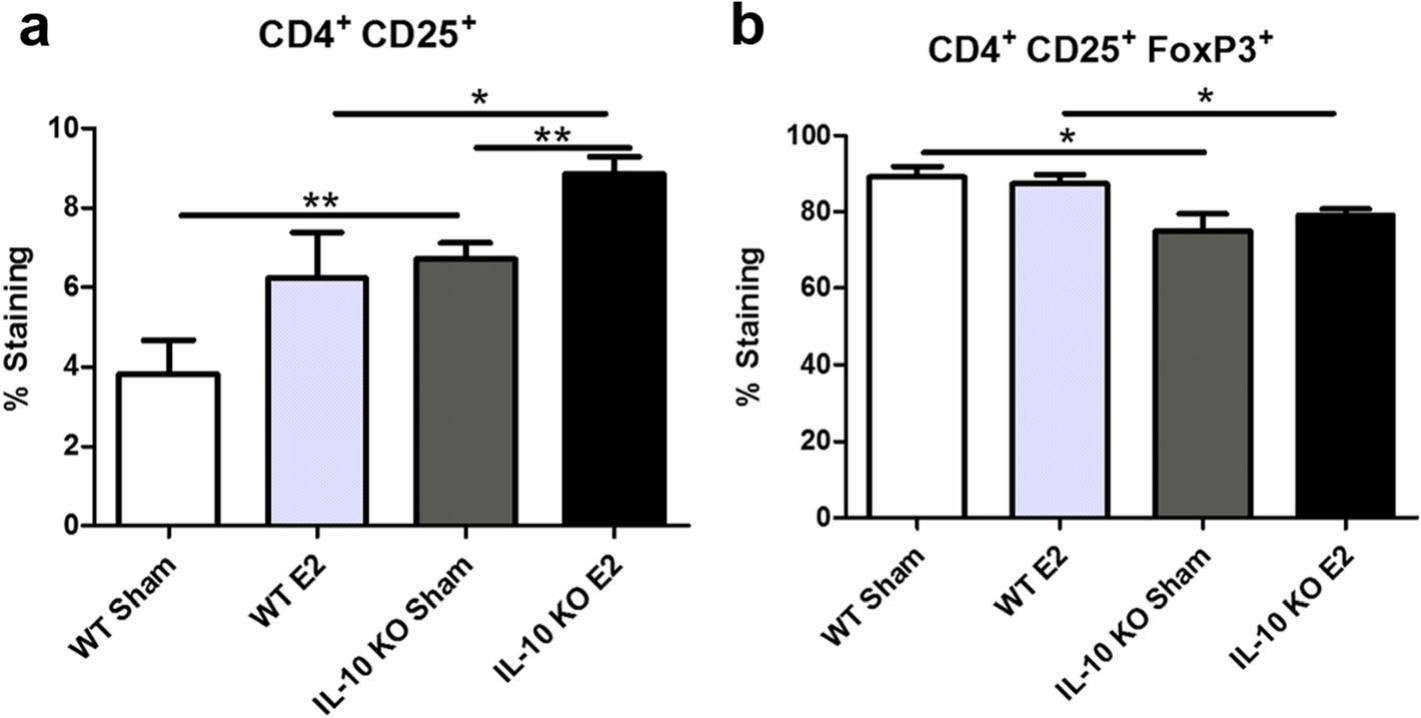
Activated T cells and FoxP3 Treg cell number are significantly different in IL-10 KO mice. The frequency of activated T cells (CD4^+^CD25^+^) in the spleen was significantly increased in IL-10 KO mice (*n* = 8) in both sham and E2 treatment groups compared to WT-treated mice (*n* = 6 and *n* = 8, respectively; *p* < 0.01). Additionally, E2-treated IL-10 KO mice had significantly more activated T cells compared to IL-10 KO sham mice (*p* < 0.05; **a**). Conversely, CD4^+^CD25^+^FoxP3^+^ Treg frequency was significantly decreased in IL-10 KO mice in both treatment groups compared to WT mice (*p* < 0.05; **b**). Data are represented as mean ± SEM

The frequency of activated CD4^+^CD25^+^ splenic Th cells was significantly increased in IL-10 KO mice compared to their respective WT treatment groups in both E2- and sham-treated groups (Fig. 4a). However, the frequency of CD4^+^CD25^+^FoxP3^+^ regulatory T cells was significantly decreased in the spleens of IL-10 KO mice compared to WT mice in both the E2-treated and sham-treated groups (Fig. 4b).

### Decreased resting microglia and increased lymphocytes in the CNS of IL-10 KO mice with EAE

**Fig. 5:**
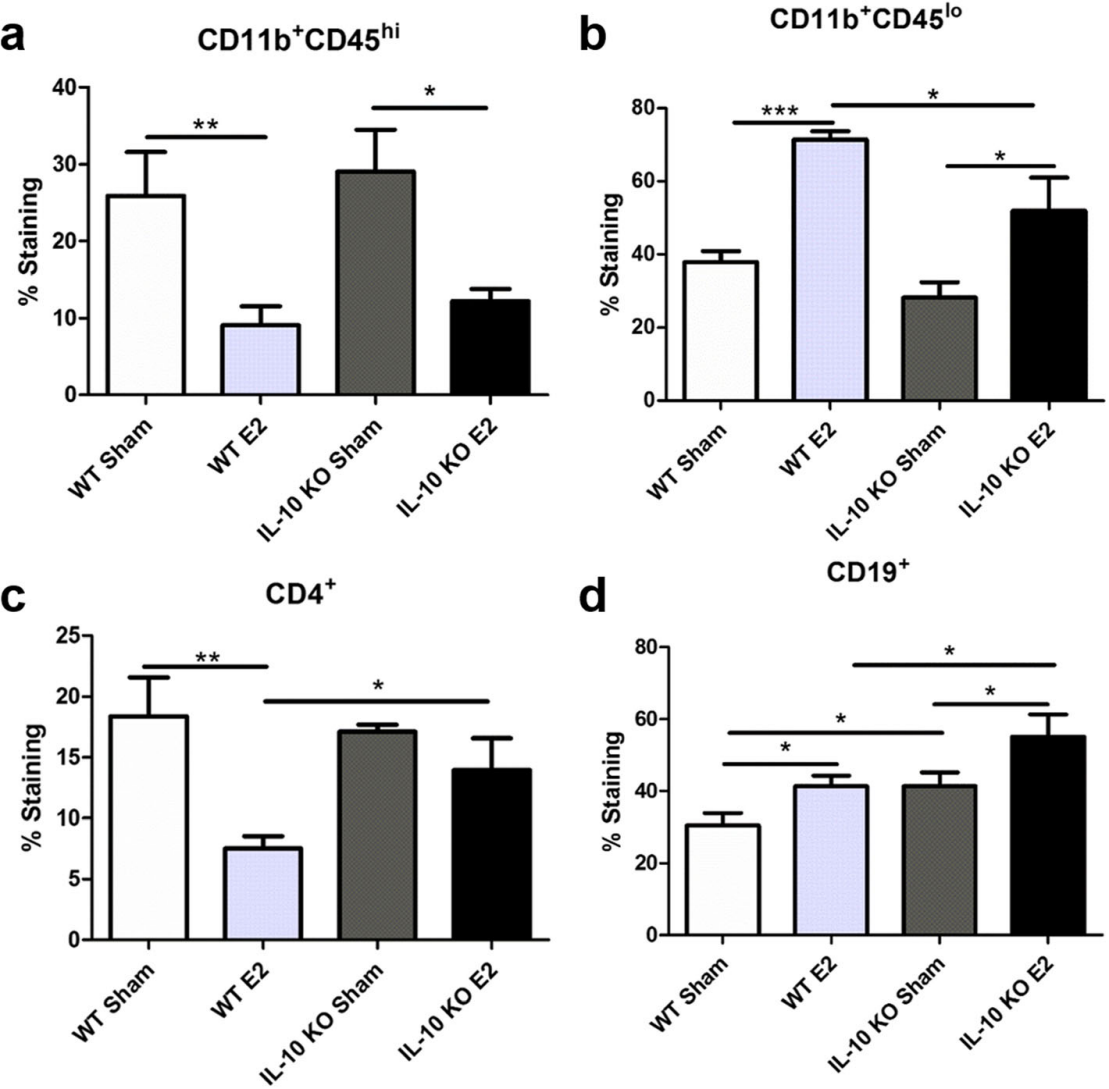
Decreased resting microglia and increased lymphocytes in IL-10 KO mice brains. Within the brain the frequency of activated (CD11b^+^CD45^hi^) microglia/macrophages was significantly decreased after E2 treatment in both WT (*p* < 0.01) and IL-10 KO (*p* < 0.05) mice compared to sham-treated mice (**a**). E2 treatment significantly increased the frequency of resting (CD11b^+^CD45^lo^) microglia in WT (*p* < 0.001) and IL-10 KO (*p* < 0.05) compared to sham-treated mice. IL-10 KO mice treated with E2 also had a significantly decreased frequency of resting microglia compared to WT E2 mice (*p* < 0.05; **b**). In WT mice (*n* = 8), E2 treatment significantly decreased the frequency of CD4^+^ cells compared to sham-treated mice (*n* = 6; *p* < 0.01). IL-10 KO E2 mice had a significantly increased frequency of CD4^+^ cells compared to WT E2 mice (*p* < 0.05; **c**). The frequency of CD19^+^ B cells was significantly increased with E2 treatment compared to sham-treated mice in both WT and IL-10 KO mice (*p* < 0.05). IL-10 KO mice also had a significantly increased frequency of CD19^+^ B cells compared to WT mice in the E2- and sham-treated groups (*p* < 0.05), with the IL-10 E2-treated group having the highest frequency (**d**). Data are represented as mean ± SEM

Microglia/macrophage activation and lymphocyte invasion in the brains of mice with EAE play a role in disease severity. Treatment with E2 significantly decreased the frequency of activated microglia/macrophages (CD11b^+^CD45^hi^) in the brains of both WT and IL-10 KO mice compared to sham-treated mice (Fig. 5a). Conversely, E2 treatment significantly increased the frequency of resting microglia (CD11b^+^CD45^lo^) in the brains of both WT and, to a lesser degree, IL-10 KO mice compared to sham-treated mice (Fig. 5b). In addition to decreasing microglia/macrophage activation, treatment with E2 also significantly decreased the frequency of CD4^+^ T cells in WT mouse brains compared to sham-treated WT mice. However, E2 treatment significantly increased the frequency of CD4^+^ cells in the brains of E2-treated IL-10 KO mice compared to E2-treated WT mice (Fig. 5c). Moreover, E2 treatment significantly increased the frequency of CD19^+^ B cells in the brains of WT and IL-10 KO mice. Of note, IL-10 KO mice significantly elevated the baseline frequency of CD19^+^ B cells in the brain compared to WT mice, with an even further increased level of B cells present after E2 treatment, compared to the E2-treated WT group (Fig. 5d).

### E2 prevents demyelination and reduces nucleated cells in the white matter

**Fig. 6:**
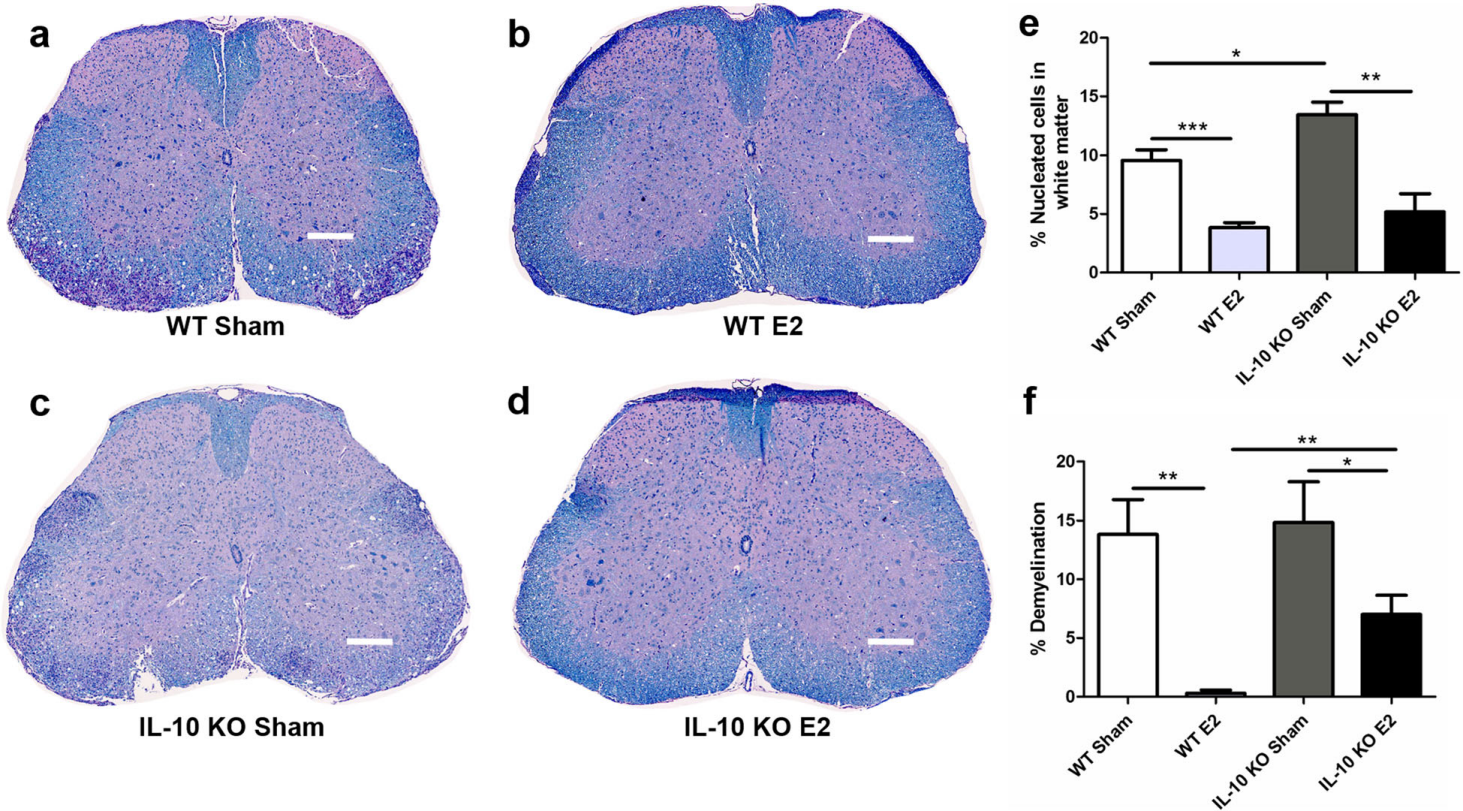
E2 treatment decreases nucleated cells in spinal cord white matter and decreases demyelination. Spinal cord sections were stained with Luxol Fast Blue/periodic acid-Schiff/hematoxylin and analyzed for the percentage of nucleated cells in the white matter and for demyelination. A representative section from WT sham (*n* = 4) mouse shows purple areas of nucleated cells and loss of myelin, indicated by blue staining (**a**). In the WT E2-treated (*n* = 4) mouse, there was an intact myelin and no dense areas of purple, nucleated cells in the white matter, demonstrated by the intact blue staining (**b**). The IL-10 KO sham (*n* = 4) mouse had several concentrated areas of purple, nucleated cells in the white matter and a loss of most of the blue staining indicating diffuse demyelination (**c**). The IL-10 KO E2 (*n* = 4) mouse had a few small areas of purple nucleated cells in the white matter and minimal myelin loss (**d**). Scale bar equals 100 μm. A quantitation of the percentage of nucleated cells within the white matter in each group showed E2 treatment significantly decreased the percentage of nucleated cells in the white matter compared to the sham treatment groups for both the WT and IL-10 KO mice (*p* < 0.01). There was also a significant increase in the percentage of nucleated cells in the white matter of the IL-10 KO sham compared to the WT sham (*p* < 0.05; **e**). E2 treatment significantly decreased the percentage of demyelination in WT and IL-10 KO mice (*p* < 0.05). There was also a significant increase in the percentage of demyelination in the IL-10 KO E2 mice compared to the WT E2 mice (*p* < 0.01; **f**). Data are represented as mean ± SEM

The lumbar section of spinal cords from mice 21 days post-immunization was stained with Luxol Fast Blue/periodic acid-Schiff/hematoxylin and imaged using light microscopy. Representative images show demyelination and infiltrating nucleated cells in the white matter of sham-treated WT (Fig. 6a) that are significantly reduced after E2 treatment (Fig. 6b). Similar to WT mice, lumbar sections from sham-treated IL-10 KO mice also display large areas of demyelination and increased nucleated cells in the white matter (Fig. 6c), with decreased areas of demyelination and decreased nucleated cells in the white matter after E2 treatment (Fig. 6d). Further analyses of the sections indicate that the IL-10 KO sham mice had significantly more infiltrating cells into myelinated areas than WT sham mice and that E2 treatment resulted in a significant reduction in the percentage of nucleated cells in the white matter of both WT and IL-10 KO mice (Fig. 6e). Moreover, treatment with E2 significantly decreased the percentage of demyelination within the white matter in both WT and IL-10 KO mice, although there remained a significantly higher percentage of demyelination in the E2-treated IL-10 KO mice compared to the E2-treated WT mice (Fig. 6f).

### Downregulation of pro-inflammatory cytokines, chemokines, and their receptors in spinal cords of E2-treated IL-10 KO mice protected from EAE

**Fig. 7:**
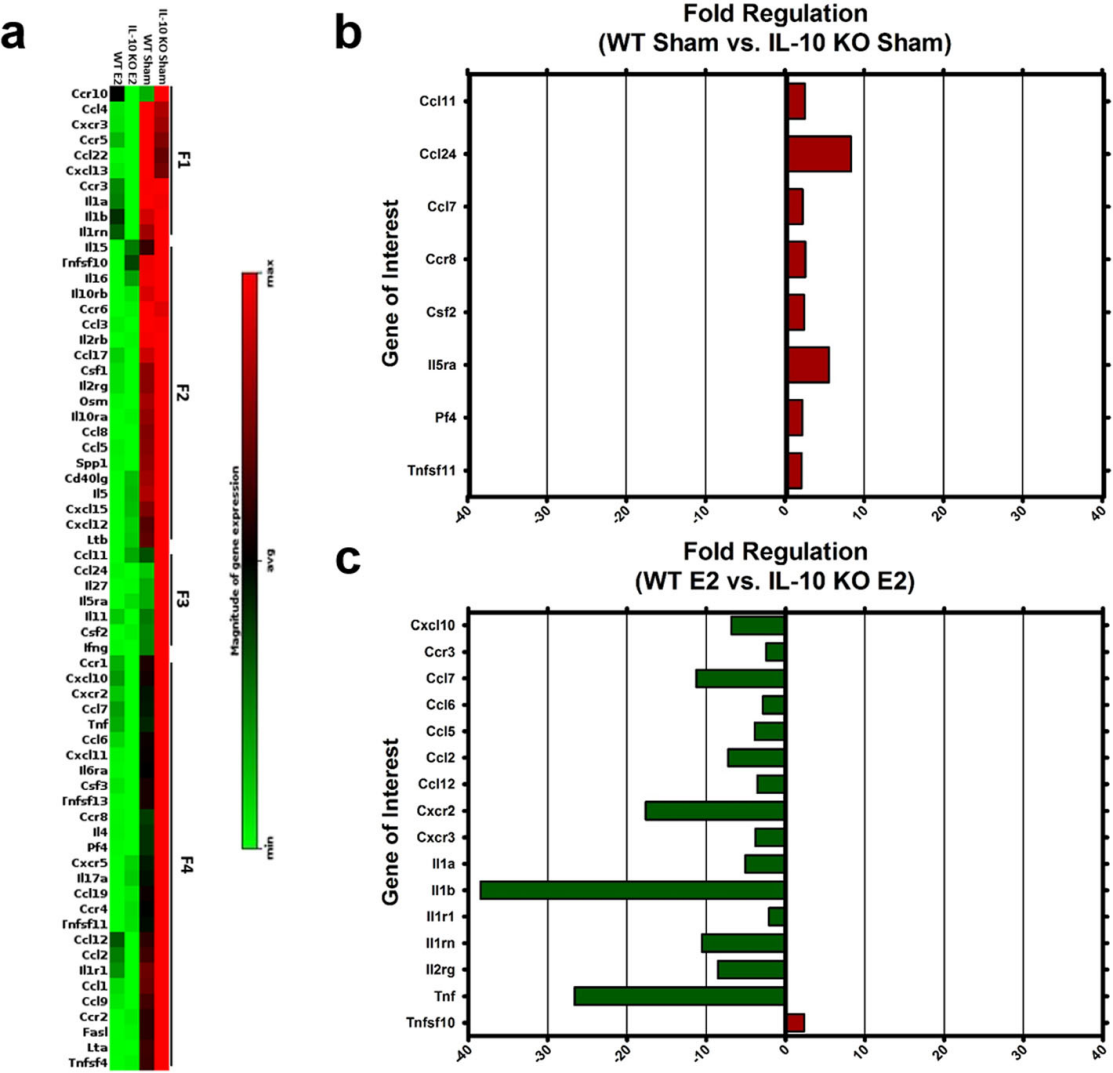
IL-10 KO mice treated with E2 downregulate inflammatory cytokines, chemokines, and chemokine receptors in the spinal cord. The two E2-treated groups, WT (*n* = 3) and IL-10 KO (*n* = 3), tend to downregulate most of the inflammatory cytokines, chemokines, and their receptors, where the sham groups, WT (*n* = 3) and IL-10 KO (*n* = 3), tend to upregulate these same genes as shown in the cluster gram of significantly regulated genes (*p* < 0.05 and fold regulation of 0–2). High-throughput analysis clustered genes on the consensus of fold regulation changes into four families of genes, F1–F4 (**a**). When looking at only the WT sham group compared to the IL-10 KO sham group, eight genes were significantly upregulated in the IL-10 KO sham group (**b**). Comparing the WT E2 group to the IL-10 KO E2 group, the IL-10 KO E2 significantly downregulated 15 key inflammatory cytokines, chemokines, or receptors and significantly upregulated one gene compared to the WT E2 group (**c**). Data are represented as mean ± SEM

The expression of cytokines and chemokines known to attract specific types of immune cells was measured in the spinal cords of sham- and E2-treated WT vs IL-10 KO mice. To determine which factors played a role in protecting mice with E2 in an IL-10-independent manner, a panel of inflammatory cytokines, chemokines, and their receptors were evaluated to determine expression levels in the spinal cord tissue. Comparison of the four treatment groups demonstrated that, whereas sham treatment of both WT and IL-10 KO mice generally upregulated inflammatory genes, treatment with E2 generally downregulated the same genes (Fig. 7a). Further analysis revealed eight genes (*Ccr8*, *Csf2*, *Il-15rα*, *Cxcl4*, and *Tnfsf11*, *Ccl11*, *Ccl24*, and *Ccl7*) that were significantly upregulated in IL-10 KO sham mice compared to WT sham (Fig. 7b).

In contrast, spinal cord tissue from E2-treated WT and IL-10 KO mice regulated inflammatory genes in significantly different ways. Compared to E2-treated WT mice, E2-treated IL-10 KO mice significantly downregulated 15 genes and significantly upregulated only one gene. All the downregulated genes are *Cxcl10*, *Ccr3*, *Ccl7*, *Ccl2*, *Ccl12*, *Cxcr3*, *Ccl5*, *Ccl6*, *Cxcr2*, *Cd134* (*Ox40*), *Il-1α*, *Il-1β*, *Il-1r1*, and *Il-1rn*. *Tnf* is also downregulated with E2 treatment in IL-10 KO vs WT mice, consistent with previously published data [8]. The one gene that is significantly upregulated in IL-10 KO compared to WT mice treated with E2 is *Tnfsf10* (Fig. 7c).

## Discussion

IL-10 is an important immunoregulatory cytokine in EAE and MS. The lack of IL-10 (in IL-10 KO mice) results in severe EAE, whereas overexpression of IL-10 can protect mice from developing EAE [33, 34]. Most Breg populations secrete IL-10 as a mechanism to mitigate immune responses [47]. These same Breg cell populations have been shown to protect mice from developing EAE [28–30]. Experiments with E2, which protects mice from developing EAE [48], have demonstrated the upregulation of IL-10-producing Breg cells [43]. Despite the data pointing to IL-10 as having an important role in E2-mediated protection from EAE, IL-10 KO mice are protected clinically and histologically from EAE when treated with E2, similar to WT mice treated with E2. These findings are consistent with previous data from our lab. We also found E2 significantly downregulates TNF production [8] in both WT and IL-10 KO mice but that this inhibition was more pronounced in IL-10 KO mice than in WT mice. In addition to IL-10, PD-L1 was also shown to play an important role in E2-mediated protection, as PD-L1 KO mice were not protected by E2 from EAE [43]. B cell-deficient μMT KO mice are also not protected by E2 [49], but protection can be restored after the transfer of IL-10-producing B cells [24]. However, IL-10-producing B cells only partially restore protection in PD-L1 KO mice [27].

These data prompted our examination of the role of the PD-L1 and PD-L2 pathways in E2-mediated protection of IL-10 KO mice. E2 significantly upregulated PD-L1 and PD-L2 on CD11b^+^ cells in the spleen, even though the activation states of CD11b^+^ cells in the spleen were similar in all groups (Additional file 1: Figure S1). In the inguinal lymph nodes, there was an increase in the frequency of PD-L2 on CD11b^+^ cells and PD-L2 was significantly increased within the spinal cords of E2-treated IL-10 KO mice compared to WT E2-treated mice. These data suggest that PD-L1 and PD-L2 may be checkpoint inhibitors induced by E2 in IL-10 KO mice that mediate protection against EAE. An additional study showed PD-L1 expression by microglia can inhibit Th_1_ cells in the CNS during EAE [41]. However, there was no reported effect on PD-1 expression.

IL-10 KO mice treated with E2 had a significantly increased number of CD73^+^ Breg cells in the spleen. B10 (CD19^+^CD5^+^CD1d^hi^) and plasmablast (CD19^+^CD138^+^CD44^hi^) subsets both had significantly more CD73-expressing cells in the spleens of E2-treated IL-10 KO mice compared to WT E2-treated mice. This increase in CD73 was found only on Breg cell subpopulations and not the total B cells (CD19^+^ population) in the spleen (Additional file 2: Figure S2). Previous studies demonstrated that CD73 KO mice are protected from developing EAE as CD73 enables immune cells to enter the CNS [50]. This mechanism is thought to be mediated through CD73^+^ cell secreted adenosine that increased CX3CL1 expression in the choroid plexus [51]. CD73-expressing Bregs have been found in mouse models of colitis, and the adenosine produced by CD73 on these Bregs contributed to the resolution of the colitis [46]. The increased expression of CD73 on Breg cell subsets in the spleens of IL-10 KO mice suggests these cells are contributing to trying to decrease inflammation in these mice to compensate for the loss of IL-10, as both the sham- and E2-treated IL-10 KO mice had significantly more CD73^+^ Breg subsets compared with WT mice. The E2-dependent increase in the expression of CD73 in the spinal cord only found in the IL-10 KO mice suggests CD73 is contributing to the protective immune response in these mice that allows them to remain asymptomatic similar to WT mice.

In addition to Bregs, E2 treatment can also affect CD4^+^CD25^+^ Tregs which include those expressing FoxP3 [52, 53]. E2-treated IL-10 KO mice had significantly more activated CD4^+^ T cells, but significantly fewer CD4^+^CD25^+^FoxP3^+^ cells in the spleen compared to WT E2-treated mice. There were no significant differences in the frequencies of Th_1_ (CD4^+^T-bet^+^) or Th_17_ (CD4^+^RORγ^+^) cells in the spleen or the inguinal lymph nodes (Additional file 3: Figure S3). There were also no significant differences in the expression of interferon gamma or IL-17 in the spinal cords (data not shown). These data suggest IL-10 KO mice either do not have as many splenic Tregs as WT mice or that the Tregs in IL-10 KO mice have migrated into the CNS. Within the CNS, there are significantly fewer areas of demyelination in the E2-treated IL-10 KO mice compared to sham-treated IL-10 KO mice. The presence of infiltrating Tregs could be one mechanism by which IL-10 KO E2-treated mice control CNS inflammation to remain protected from EAE.

IL-10 KO mice treated with E2 also had a significant increase in T cells in the brain compared to WT E2-treated mice, in which E2 significantly reduced T cell numbers compared to sham-treated WT mice which is consistent with previous data [43]. These T cells would include CD4^+^CD25^+^ Tregs because these mice are protected from EAE, symptomatically and histologically. Bregs are also likely involved in protecting the CNS, since E2 increased B cells in the brain in WT mice and the IL-10 KO mice had an increased percentage of B cells in the brain compared to WT mice. Moreover, E2 treatment significantly decreased the percentage of activated microglia (CD11b^+^CD45^hi^) in both the WT and IL-10 KO mice with EAE. However, E2 treatment only modestly increased the percentage of resting microglia (CD11b^+^CD45^lo^) in IL-10 KO compared to WT mice. One possible explanation of the significantly increased percentage of demyelination in the E2-treated IL-10 KO mice compared to WT E2-treated mice is the suboptimal activation of resting microglia. That being said, it is important to note that the IL-10 KO mice remained symptom-free.

E2-induced regulation of cytokines, chemokines, and their receptors in the spinal cord also contributed to EAE protection of IL-10 KO mice. When comparing sham-treated WT vs IL-10 KO mice, the IL-10 KO mice significantly upregulated CCL7. Conversely, the E2-treated IL-10 KO mice significantly downregulated *Ccl7* compared to sham-treated WT mice. CCL7 regulates monocyte recruitment by glial cells and the expression of CCL7 is controlled by estrogen [54, 55]. CCL7 binds to CCR3, and CCR3 binds multiple other chemokines, including CCL11, CCL24, CCL13, CCL26, and CCL5 [56]. In sham-treated IL-10 KO vs sham-treated WT mice, the CCR3 ligands CCL11, CCL7, and CCL24 were significantly upregulated, and the *Ccr3* receptor was equally upregulated in both sham groups compared to E2 treatment. In IL-10 KO mice treated with E2 compared to WT E2-treated mice, the gene expression levels of *Ccr3* are significantly downregulated, effectively providing no binding site, CCR3, for the above listed pro-inflammatory chemokines, thus short-circuiting that inflammatory cascade. Another highly pro-inflammatory axis significantly affected by E2 treatment in IL-10 KO mice is the CXCL10/CXCR3 IFNγ-induced pathway, which was significantly downregulated in the E2-treated IL-10 KO mice compared to WT E2-treated mice. CXCL10 is associated with increased infiltration of immune cells across the blood-brain barrier [57] and is highly expressed in EAE [58] and stroke [59]. CXCR3 binds CXCL10 [60] and is associated with demyelination and microglial activation [61]. Another IFNγ driven pro-inflammatory chemokine, CCL6, is constitutively expressed by microglia [62] and is significantly downregulated in E2-treated IL-10 KO mice compared to E2-treated WT mice. Two other pro-inflammatory cytokines, TNF and IL-1 (both IL-1α and IL-1β), were significantly decreased in E2-treated IL-10 KO mice compared to WT E2-treated mice. E2 treatment was already shown to decrease TNF [8], but we here demonstrated even greater downregulation of TNF in E2-treated IL-10 KO compared to WT mice. This decrease in TNF is likely mediated in part by the significant downregulation of IL-1α and IL-1β which can upregulate TNF production [63] and are associated with EAE disease severity [64].

Taken together, three major compensatory mechanisms stimulated by E2 treatment of IL-10 KO mice that differ from E2 treatment of WT mice include (1) significantly greater expression of the PD-L1 and PD-L2 inhibitory checkpoint pathway on macrophages in both the periphery and CNS, (2) increased CD73 expression in the CNS tissue for local suppression through adenosine production, and (3) of the decrease in CD4^+^CD25^+^FoxP3^+^ Treg cells in the spleen taken with increased T cells in the brain and decreased spinal cord damage suggest these cells could be leaving the spleen to enter the CNS. These compensatory factors effectively inhibit the expanded array of pro-inflammatory cytokines and chemokines that drive the development of severe EAE in the absence of IL-10.

## Conclusion

**Fig. 8:**
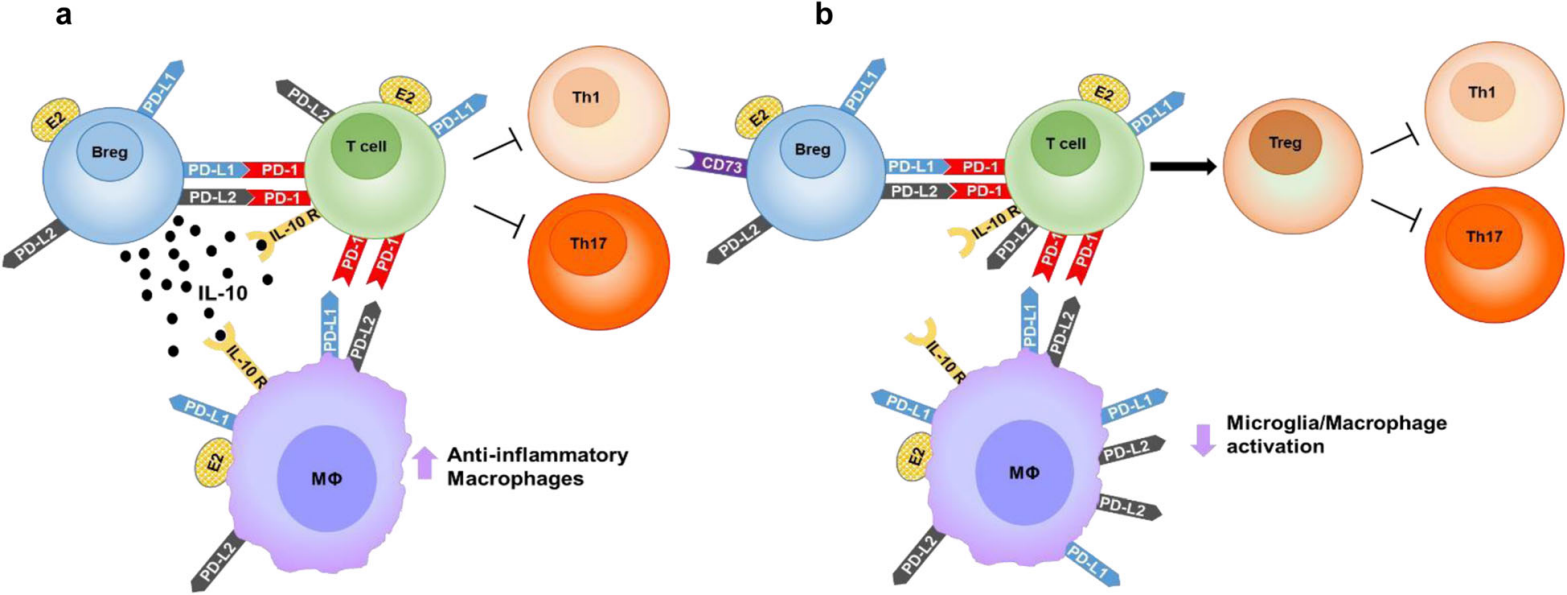
Differential E2-mediated mechanisms of EAE protection in IL-10 KO vs WT mice. Treatment with E2 in WT mice results in the induction of IL-10-producing regulatory B cells (Bregs) that modulate T cells and macrophages through the secretion of IL-10 and the activation of PD-1 on T cells. This results in a decrease in pro-inflammatory Th_1_ and Th_17_ T cells and an increase in anti-inflammatory macrophages and microglia. Treatment with E2 increases PD-L1 and PD-L2 expression on macrophages. In the CNS, E2 decreases the frequency of CD11b^+^CD45^hi^ cells and increases the frequency of CD11b^+^CD45^lo^ microglial cells during EAE compared to sham-treated mice (**a**). Treatment with E2 in IL-10 KO mice results in an increase in the number of PD-L1 and PD-L2 positive macrophages compared to WT E2-treated mice. This results in the induction of activated CD4^+^CD25^+^ splenic T cells, an increase in Breg B10 and plasmablast Breg subsets expressing CD73, a marker for an IL-10 independent Breg immunoregulatory mechanism, an increase in CNS microglial cells, and a decrease in the frequency of Th_1_ and Th_17_ pro-inflammatory cells. This results in decreased levels of CD11b^+^CD45^hi^ activated macrophages similar to WT E2-treated mice and a downregulation of pro-inflammatory mediators (**b**)

IL-10 KO mice have previously been shown to be highly protected from EAE by E2, similar to WT mice. In the previous study, the only protective mechanism identified was a decrease in TNF in all mice treated with E2. Our current study points to three alternative mechanisms impacting IL-10 KO mice when treated with E2, which maintain protection from EAE, driven by an alternative array of pro-inflammatory cytokines and chemokines: (1) increased expression of PD-L1 and PD-L2 on monocyte/macrophage cells in the periphery and the CNS, (2) an increase in CD73 in the CNS to increase the anti-inflammatory molecule adenosine, and (3) a decrease in CD4^+^CD25^+^FoxP3^+^ regulatory T cells in the spleen corresponding to a possible increase in FoxP3^+^ regulatory T cells CNS tissues. These mechanisms in IL-10 KO mice downregulate several pro-inflammatory cytokines and chemokines previously unknown to contribute to EAE in WT mice (Fig. 8). From these studies, it would appear that E2-induced IL-10 modulates inflammation in EAE mainly in the periphery of WT mice, whereas in IL-10-deficient mice, E2 induced PD-L2, Breg, and Treg cells to modulate more complex inflammatory responses mainly in the CNS. Future studies will further evaluate the role of estrogen on neural cells which may also contribute to the EAE protection seen with E2.

## Supplementary information

**Additional file 1: Figure S1.** Similar increases in macrophage activation with E2 treatment in WT and IL-10 KO mice. There was no change in Arg1 expression in CD11b^+^ cells  among the groups though there was a trend towards an increase in the E2 treated groups (a). There were significant increases in CD206 expressing CD11b^+^ cells  in both E2-treated groups (*p* < 0.001) that was not significantly different from WT or IL-10 KO (b). Data are represented as mean ± SEM.

**Additional file 2: Figure S2.** CD73 expression is unchanged on B cells. There was no significant difference in the expression of CD73 on CD19^+^ B cells among all groups. Data are represented as mean ± SEM.

**Additional file 3: Figure S3.** There is no change in the number of Th_1_ or Th_17_ cells in the spleen. There was no significant difference in the number of Th_1_ cells (a) or Th_17_ cells in the spleen (b) between any of the groups. Data are represented as mean ± SEM.

## Data Availability

The datasets used and/or analyzed during the current study are available from the corresponding author on reasonable request.

## Abbreviations

7AAD: 7-Amino-actinomycin D
APC: Antigen-presenting cells
Breg: Regulatory B cells
CDI: Cumulative disease index
E2: 17β-estradiol
EAE: Experimental autoimmune encephalomyelitis
ERα: Estrogen receptor alpha
i.p.: Intraperitoneal
IFA: Incomplete Freund’s adjuvant
IFNβ: Interferon beta 1 beta
IFNγ: Interferon gamma
IL-10: Interleukin-10
KO: Knockout
LN: Lymph nodes
MHC: Major histocompatibility complex
MOG: Myelin oligodendrocyte glycoprotein 35–55
MS: Multiple sclerosis
Mtb: *Mycobacterium tuberculosis*
PD-1: Programmed death receptor 1
PD-L1 and PD-L2: Programmed death-ligand 1 and 2
PD-L1: Programmed death-ligand 1
Ptx: Pertussis toxin
SEM: Standard error of the mean
Th: T helper
TNF: Tumor necrosis factor
Treg: T regulatory cells
WT: Wild type
μMT−/−: B cell-deficient
