# Supplementary figures and images for: Estrogen-induced compensatory mechanisms protect IL-10-deficient mice from developing EAE
Source: J Neuroinflammation. 2019 Oct 29;16:195. doi: 10.1186/s12974-019-1588-z (PMC6821034; doi:10.1186/s12974-019-1588-z)

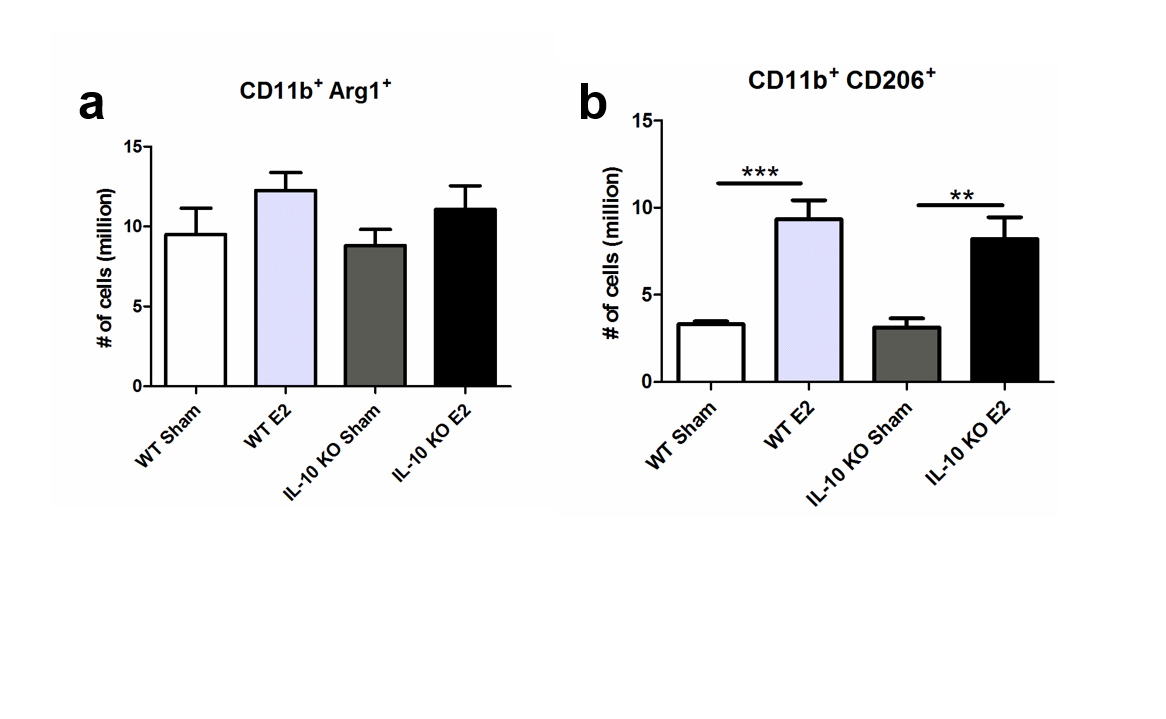

Supplement: Supplementary file 1 — Additional file 1: Figure S1. Similar increases in macrophage activation with E2 treatment in WT and IL-10 KO mice. There was no change in Arg1 expression in CD11b+ cells among the groups though there was a trend towards an increase in the E2 treated groups (a). There were significant increases in CD206 expressing CD11b+ cells in both E2-treated groups (p < 0.001) that was not significantly different from WT or IL-10 KO (b). Data are represented as mean ± SEM. [file 12974_2019_1588_MOESM1_ESM.tif]

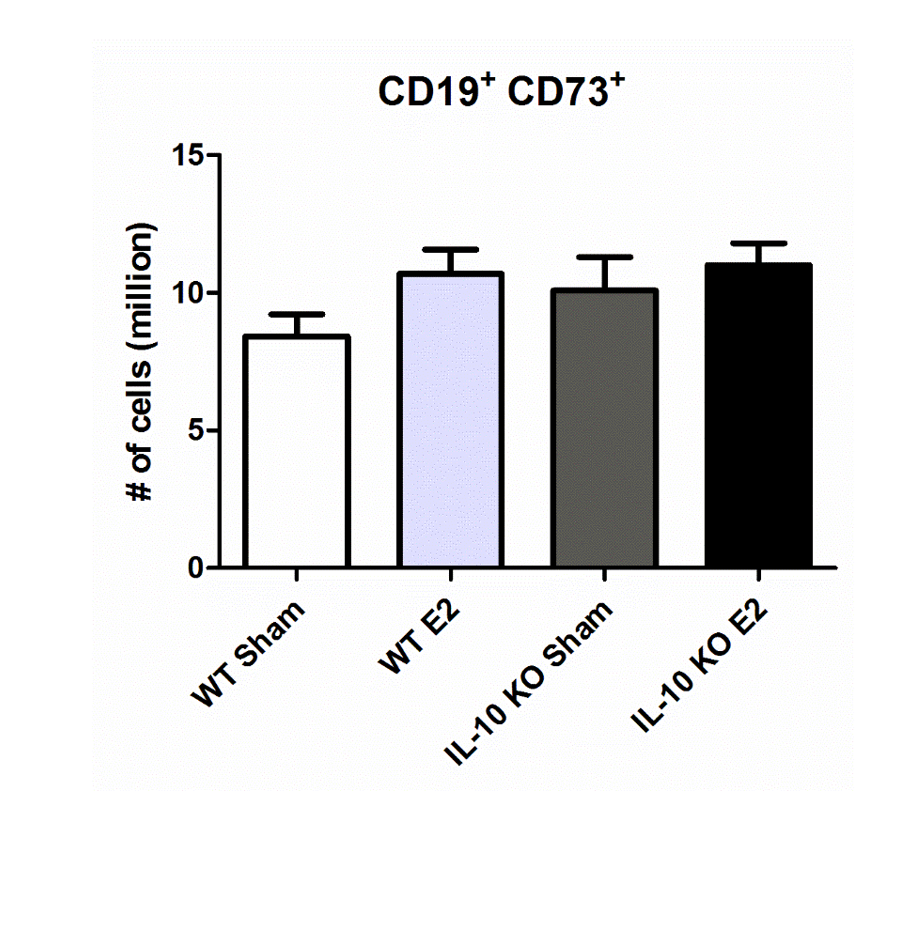

Supplement: Supplementary file 2 — Additional file 2: Figure S2. CD73 expression is unchanged on B cells. There was no significant difference in the expression of CD73 on CD19+ B cells among all groups. Data are represented as mean ± SEM. [file 12974_2019_1588_MOESM2_ESM.tif]

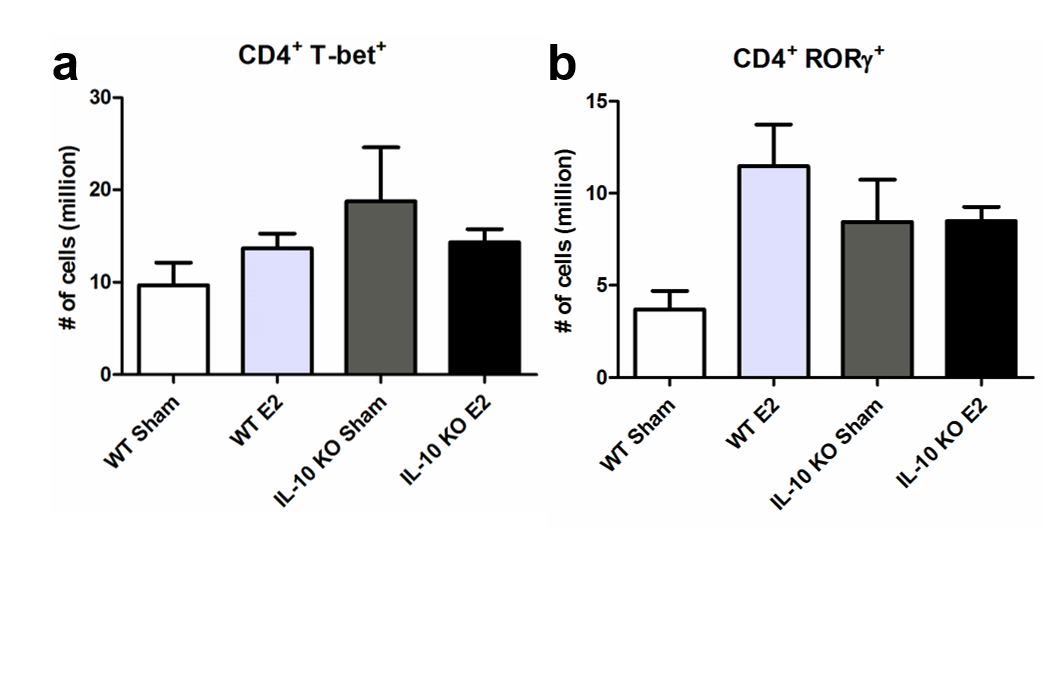

Supplement: Supplementary file 3 — Additional file 3: Figure S3. There is no change in the number of Th1 or Th17 cells in the spleen. There was no significant difference in the number of Th1 cells (a) or Th17 cells in the spleen (b) between any of the groups. Data are represented as mean ± SEM. [file 12974_2019_1588_MOESM3_ESM.tif]
